# TI nulling and contrast conditions with a 0.15 mmol/kg dosage of MultiHance as compared to the standard 0.2 mmol/kg gadolinium dosage in patients with myocardial infarcts

**DOI:** 10.1186/1532-429X-11-S1-P189

**Published:** 2009-01-28

**Authors:** June A Yamrozik, Mark Doyle, Vikas K Rathi, Diane A Vido, Ronald B Williams, Geetha Rayarao, Robert WW Biederman

**Affiliations:** Allegheny General Hospital, The Gerald McGinnis Cardiovascular Institute, Pittsburgh, PA USA

**Keywords:** Gadopentetate Dimeglumine, Gadobenate Dimeglumine, High Relaxivity, Gadolinium Dosage, Null Time

## Introduction

It is known that utilizing the proper inversion time (TI) is essential when diagnosing myocardial infarction. Many factors play a role in acquiring optimal nulling of the myocardium. Recently, higher relaxivity contrast agents have been advocated for use in infarct imaging. Specifically, MultiHance^®^ (gadobenate dimeglumine) has higher relaxivities (r_1_ and r_2,_ 9.7^1^ and 12.5^1^) compared to standard gadolinium (gadopentetate dimeglumine) (r_1_ and r_2,_ 4.9^1^ and 6.3^1^). Will this higher relaxivity allow a lower dosage of contrast to be used to maintain a comparable TI null time compared to the conventional gadolinium dosage and how does it affect contrast?

## Purpose

We hypothesize that a 0.15 mmol/kg dosage of MultiHance with its higher relaxivity will produce a lower TI null time in infarct patients compared to patients imaged with a standard dosage of 0.2 mmol/kg of gadolinium at 10 and 20 minutes post-contrast.

## Methods

A total of 52 patients with GFR > 60 mL/min/1.73 m^2^ were imaged.Twenty-six (26) patients (19 M, 6 F), age 42–81 years, post-myocardial infarction underwent a standard cardiac MRI (CMR), utilizing a total of 0.2 mmol/kg gadolinium dosage (Magnevist-Berlex, New Jersey, USA). This was administered as a 0.5 mmol/kg for perfusion imaging, followed by an additional 0.15 mmol/kg for viability (DHE) imaging. Twenty-six (26) patients (21 M, 5 F), age 43–83 years, post-myocardial infarction underwent a standard cardiac MRI (CMR), utilizing a total of 0.15 mmol/kg MultiHance dosage (Bracco Diagnostics, Princeton, N J, USA). This was administered as a 0.5 mmol /kg dosage for perfusion imaging, followed by an additional 0.10 mmol/kg for viability imaging. The scans were acquired on a GE CV/i Excite Version 12, 1.5 T system (GE, Milwaukee, WI). The sequence utilized for optimum myocardial nulling was a standard 2D Gradient Echo IRP (FGR with inversion recovery prep). Manual selection of TI was performed independent of contrast media. Contrast of infarct to myocardium and blood pool was measured in a subset of 14 patients (7 per group) with large infarcts. An 8-channel or 4-channel cardiac coil was used, again independent of the contrast agent. The sequence parameters were as follows: TE: min, FA: 20, NEX: 2 trigger delay: adjusted to onset of diastole, 1 RR interval and TI adjusted to null the myocardium. This sequence was performed at 10 and 20 minutes post-contrast administration.

## Results

All the patients successfully completed the CMR examination without difficulty, or impairment in infarct detection. Particular attention was focused on the contrast medium utilized, dosage and its effects on the TI at 10 and 20 minutes post-contrast. At 10 minutes post-contrast, the inversion time with Magnevist was 158.7 ± 17.2, which was not different from that obtained with MultiHance (154.3 ± 13.6, p = 0.3). At 20 minutes post-contrast, the TI with Magnevist was 200.4 ± 28.8, and again was not different from that obtained with MultiHance (193.0 ± 15.6, p = 0.3).

The signal intensity contrast between the infarct and myocardium at 10 minutes is greatest with the MultiHance a ratio of (7.6 ± 2.5) compared to (4.9 ± 1.2) with gadolinium.

## Conclusion

While the relaxivity of MultiHance compared to standard gadolinium is almost a factor of two higher, similar contrast null times are achieved by using a 75% dosage of MultiHance as compared to gadolinium. Relaxivity alone does not predict arithmetic decreases in TI times. Despite this, MultiHance administration at the lower dose did result in a higher infarct-myocardium contrast. Further, the higher relaxivity agent is potentially beneficial in those situations when additional imaging might be necessary, since an additional 25% of contrast is held in reserve for further administration.

